# A case report of primary cardiac angiosarcoma with *DNMT3A* gene mutation

**DOI:** 10.3389/fonc.2022.1018741

**Published:** 2022-10-27

**Authors:** Kun Tian, Yating Liu, Haiying Zhu, Tao Wang, Tianxing Chen, Xiaxia Pei, Feixue Song

**Affiliations:** Department of Medical Oncology, Second Hospital of Lanzhou University, Lanzhou, China

**Keywords:** cardiac angiosarcoma, *DNMT3A*, surgery, chemotherapy, cardiac tumor rupture

## Abstract

Cardiac angiosarcoma is a rare disease with a high mortality rate despite its low incidence. Surgery is currently the mainstay treatment strategy for patients with this condition. Herein, we describe a case of primary cardiac angiosarcoma, including symptoms, examination findings, treatment strategy and prognosis. In 2020, the patient was admitted to our hospital, and Next-Generation Sequencing (NGS) revealed a mutation in the *DNMT3A* gene. Generally, *DNMT3A* mutations are most commonly seen in atherosclerosis and myeloid leukemia. To our knowledge, this is the first reported case of primary cardiac angiosarcoma with *DNMT3A* gene mutation.

## Introduction

Primary cardiac neoplasm is extremely rare, with an incidence of 0.001%-0.003%, among which only 25% of cases are classified as malignant (1). Primary cardiac angiosarcoma (PCAS) is the most common histological subtype (50%-75%) characterized by high malignancy and invasiveness. It originates from vascular endothelial cells and tends to invade the myocardium, valves, pericardium and even the coronary arteries. At the time of diagnosis, around 89% of patients reportedly have distant metastases (2, 3). PCAS patients have a poor prognosis in general, with the median overall survival (OS) estimated at around 16 months to as low as 2 months in advanced metastatic cases (4). In this case report, we present a unique case of PCAS induced by a mutation in the DNMT3 gene.

## Case presentation

**Figure 1:**
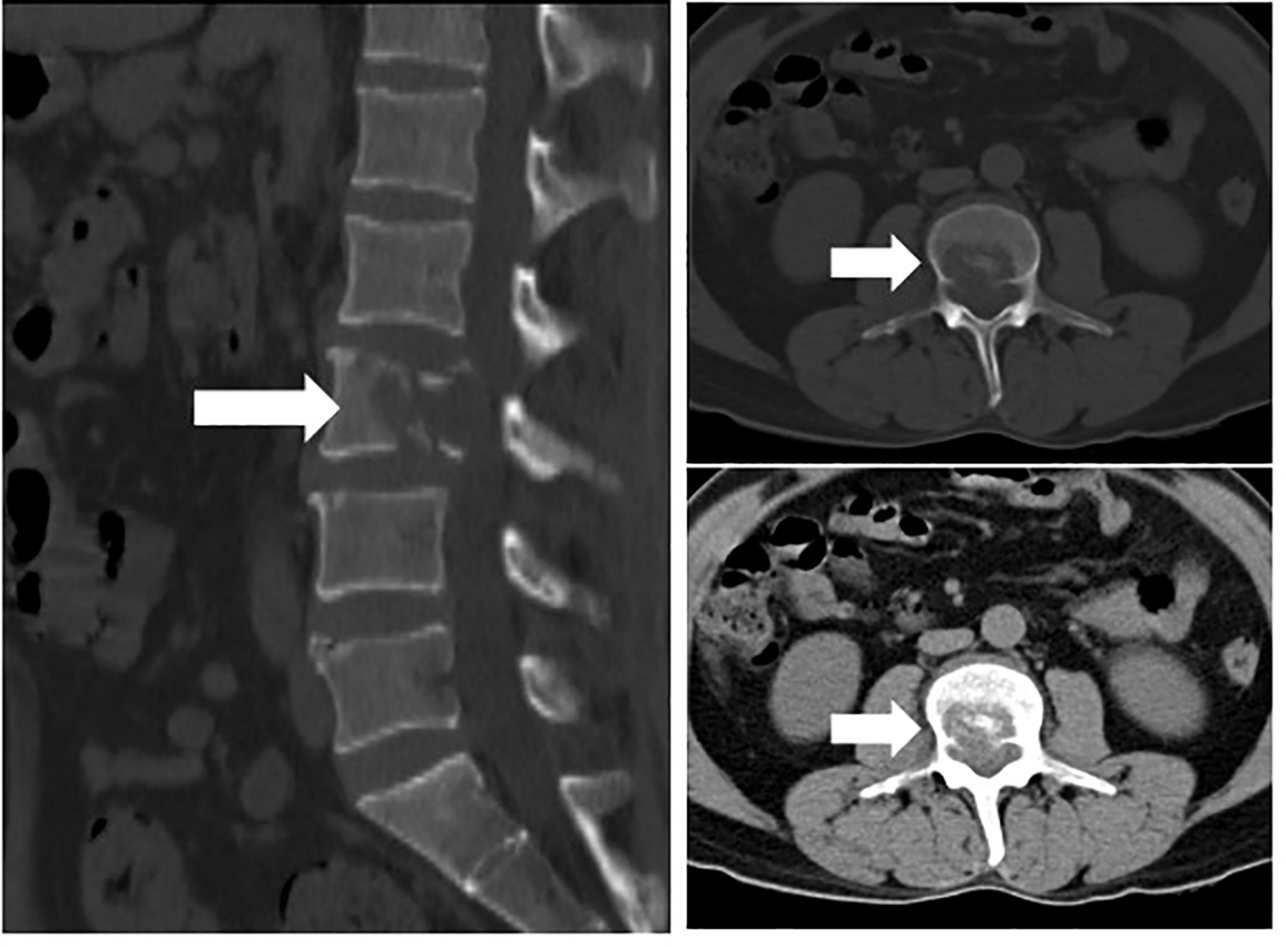
Bone destruction of the L3 vertebral body with abnormal low-density shadow within it(arrows).

**Figure 2:**
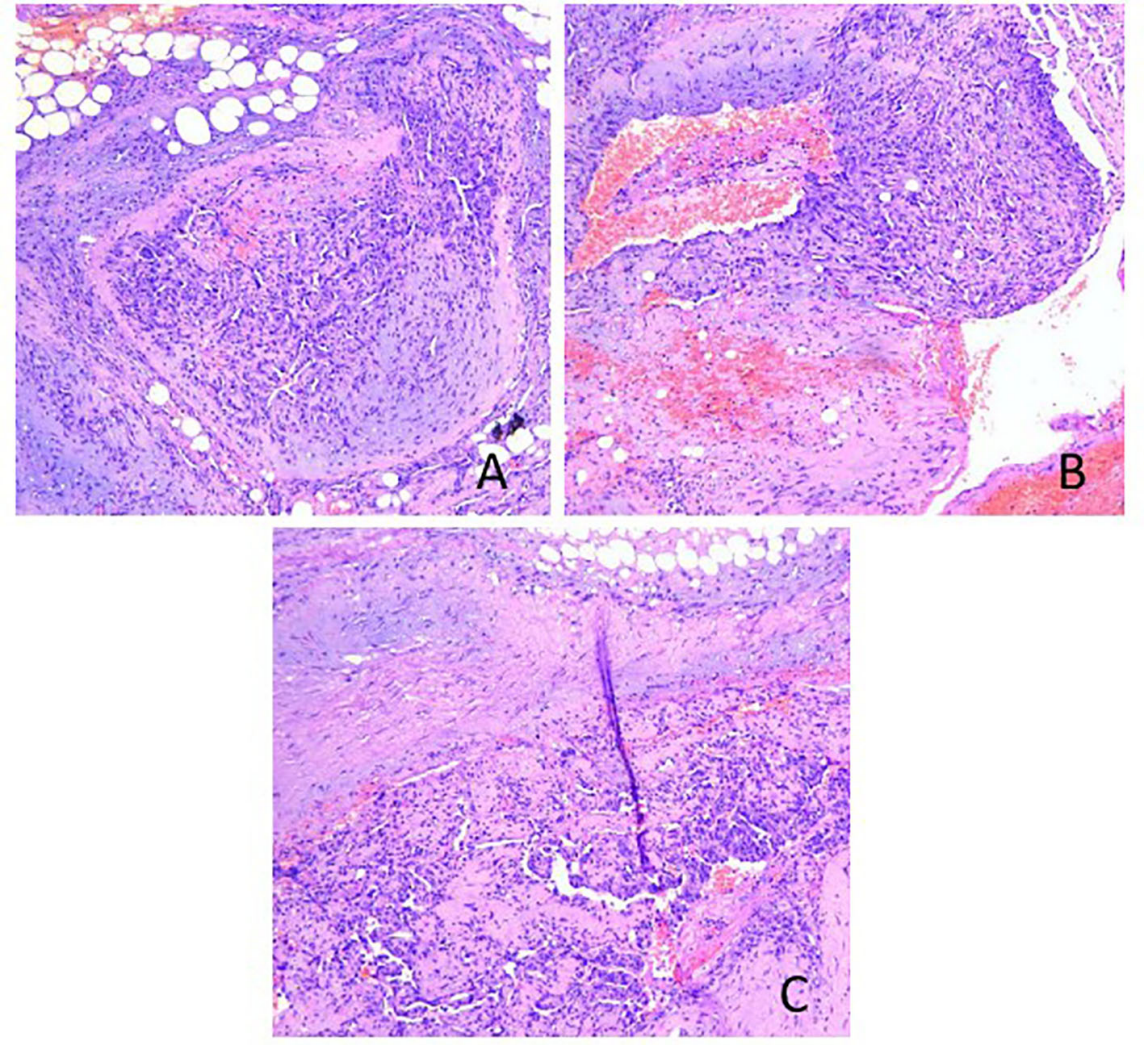
Hematoxylin & eosin staining of the L3 biopsy: **(A)** The tumor tissue consisted of well-differentiated vascular lumen, which grew infiltratively around; **(B)** the lumen was irregular in size and shape, communicating with each other to form anastomosis or communication, and erythrocytes were seen in the lumen; **(C)** the lumen was lined with monolayer, dark-stained endothelial cells, partly forming papillae in the lumen, and the tumor cells were heterogeneous with large, dark-stained, heterogeneous nuclei.

**Figure 3:**
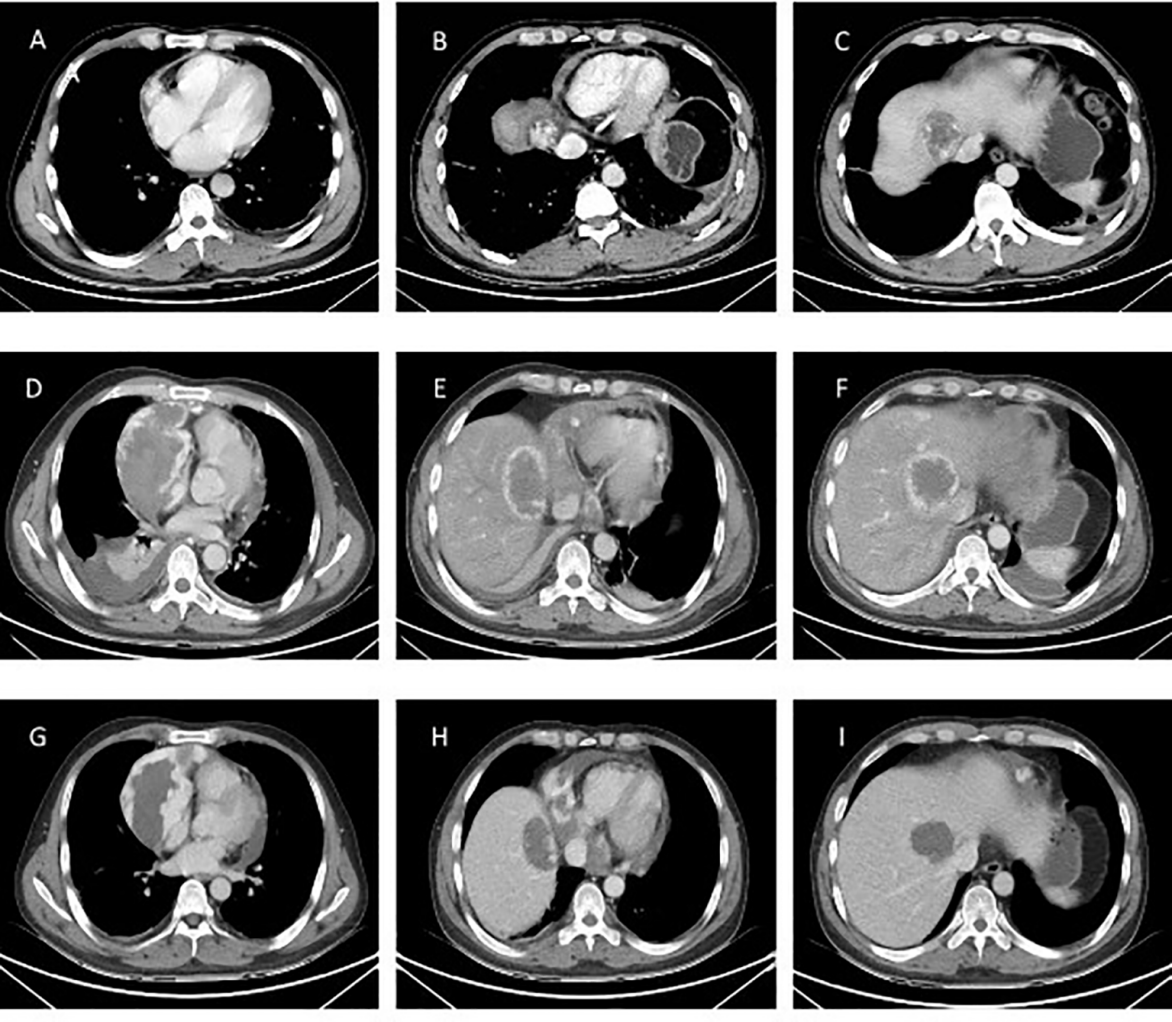
CT examination of the patient. **(A-C)** Patient’s initial CT image in March 2020. Space-occupying lesions in the right atrium, liver segment S8, pericardium; **(D-F)** CT images at the time of the patient’s readmission in May 2020. The right atrium and liver lesion were found to be significantly larger than before, as well as pericardial effusion, right pleural effusion and incomplete right lower lung distension were detected; **(G-I)** Efficacy evaluation after 1 cycle of chemotherapy with stable disease in June 2020.

The patient is a 45-year-old middle-aged male, a farmer by profession, with no history of chronic diseases and no family history of tumors. He visited the Department of Orthopedics for “lumbago for a week” in early March 2020. CTimaging revealed bone destruction at the L3 vertebral level, with an abnormally low-density shadow (See **Figure 1**). The bone scan showed low uptake, but its properties remain to be determined. A biopsy was conducted, and pathological analysis revealed angiosarcoma (L3). Immunohistochemical (IHC) staining showed the following: CD31 (+), CD34 (+), Vimentin (+), CKp (-), S-100 (-), and 30% Ki-67-positive cells (See **Figure 2**). Considering that the angiosarcoma (L3) was metastatic, a whole-body CT scan was performed. Space-occupying lesions in the right atrium, liver segment S8, pericardium and bone (See **Figure 3**). Echocardiography (ECG) revealed: EF of 60% and FS of 31% in the Left ventricular ejection fraction (LVEF); a mean pulmonary arterial pressure (mPAP) of 17 mmHg; effusion in the pericardial cavity; pericardial thickening in the lateral right ventricular wall with the widest diameter measuring about 11 mm; and a hypoechoic lesion (6.4×5.7 cm) in the right atrial roof with inhomogeneous echogenicity causing compression of the atrium. During his hospital stay, the patient experienced pericardiac tamponade. Pericardiocentesis and pericardial drainage were performed, and 600 ml of hemorrhagic fluid was extracted and screened for cancer cells using cytology. After a period of symptomatic treatment, the patient felt better and discharge.

**Figure 4:**
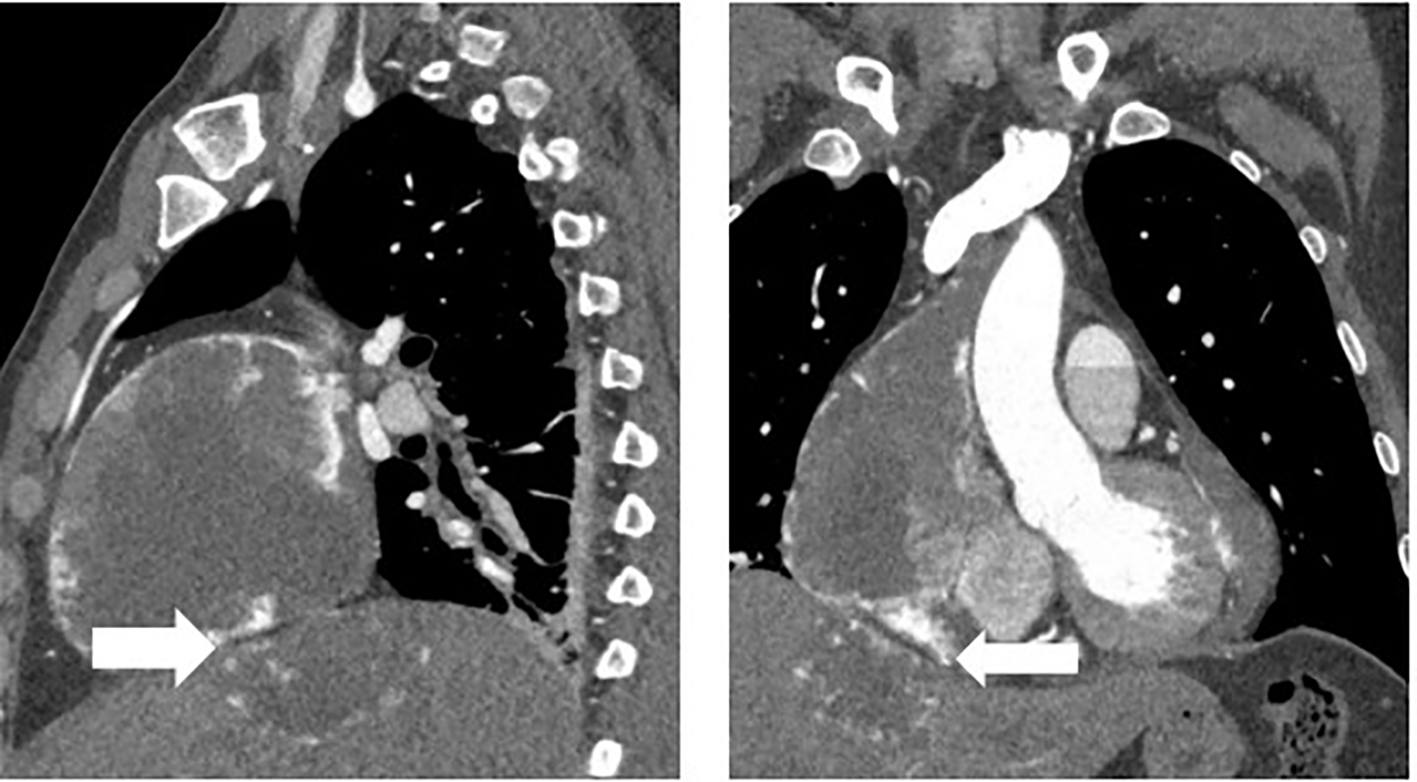
Direct invasion of the liver by a right atrial lesion(arrows).

**Figure 5:**
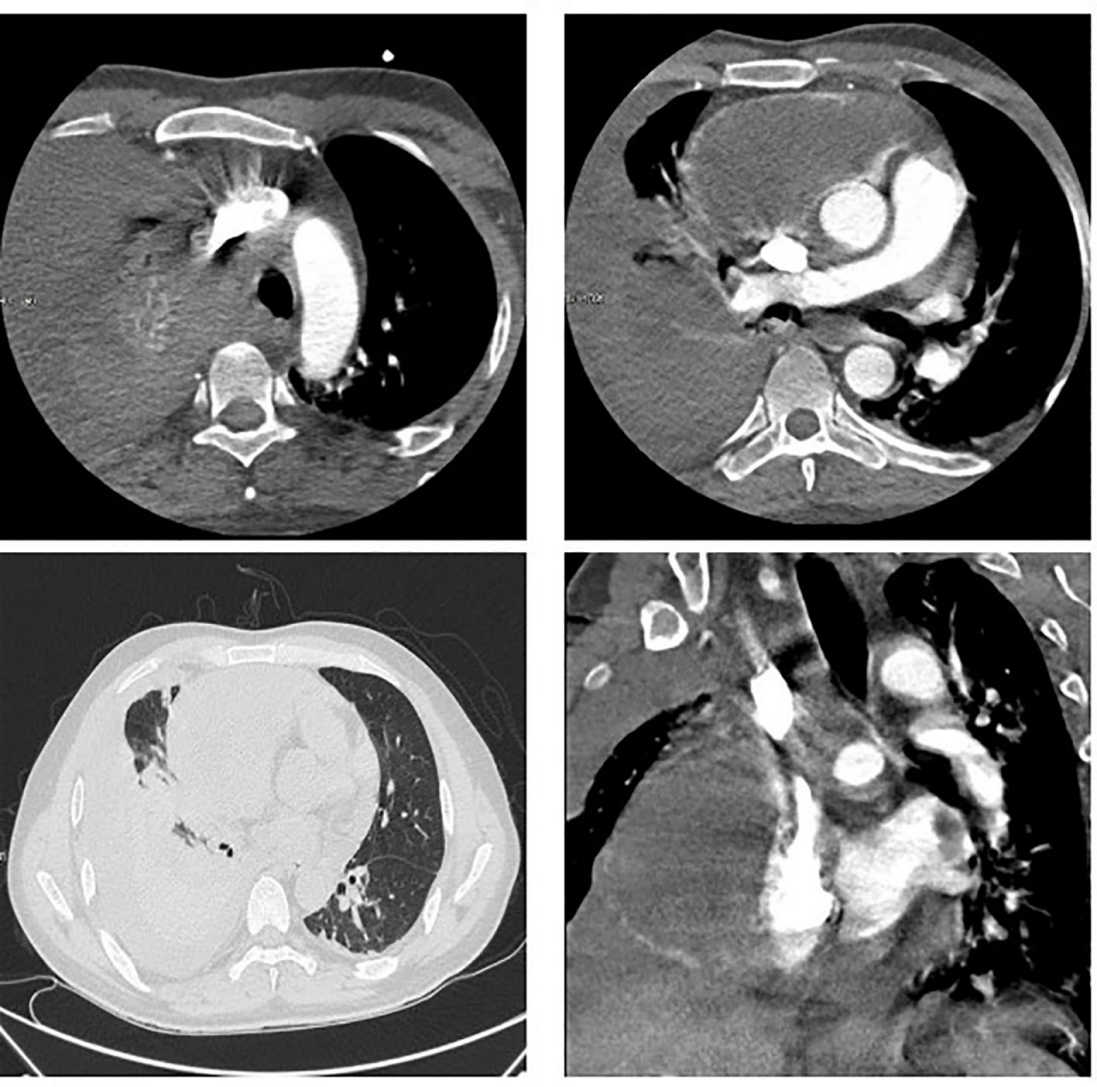
Massive pleural effusion, diagnosis of cardiac tumor rupture, but no exact bleeding point found.

The patient was readmitted to the hospital in May 2022 with chest tightness, shortness of breath, and pain in the anterior chest region. A CT examination was performed and the right atrium and liver lesion were found to be significantly larger than before, as well as pericardial effusion, right pleural effusion and incomplete right lower lung distension were detected (See **Figure 3**). It was a challenge to determine the primary focus, for which we conducted a multidisciplinary discussion, and by reviewing the literature we found that angiosarcoma with primary origin in the heart accounted for 23.5%, while the percentage of primary in the liver and vertebral body was 7.4% and 4.4%. In addition, through imaging we found that the lesion in the liver was most likely due to direct invasion of the right atrial lesion into the liver (See **Figure 4**), so we judged that the primary lesion in this patient was the right atrium. Considering the risk of rupture of the cardiac tumor at any time, the patient was advised to remove the cardiac tumor and repair the wall of the right atrium, but the patient and his family refused the surgery considering the riskiness and cost of the procedure. Before starting treatment, we performed NGS of the L3 bone puncture specimen and found a 10.7% abundance of a missense mutation in exon 19 of the DNMT3A gene (p.Glu733Ala). There is no standard treatment option for patients with advanced metastatic PCAS. Although we identified mutations in the DNMT3A gene, there are no corresponding targeted drugs; the TMB of this patient was 1.0 mutations/Mb, microsatellite stability(MSS) type, suggesting that immunotherapy may not be effective; meanwhile, the patient’s tumor progressed too fast, the radiation treatment time was long, and there was a risk of acute cardiac rupture during the treatment, and the radiotherapy department evaluated and recommended 2 cycles of chemotherapy first to evaluate the efficacy before deciding whether to give radiotherapy. Therefore, we chose Gemcitabine (1.4 g, Day 1, 8) and Docetaxel (150 mg, Day 8) for two cycles starting May 2020. The Gemcitabine dose was then increased to 1.5 g in June and after 1 cycle, stable disease (SD) was achieved on CT scan (See **Figure 3**). However, in mid-July, the patient presented with acute chest pain, with blood pressure fluctuating between 80-90 and 40-50 mmHg. CT scan indicated significant pleural effusions (See **Figure 5**), and a routine blood test revealed an RBC of 2.69*10-12/L and HGB of 76 g/L. Combining the medical history, we hypothesize that the bleeding could have been caused by cardiac tumor rupture; and the patient subsequently died three days later.

## Discussion

PCAS is a very rare malignant tumor. According to Epidemiological statistics there were 16 PCAS patients admitted to the Peking Union Medical College Hospital from January 1990 to June 2017, 40 PCAS patients at the French Sarcoma Group from 1977 to 2010, 16 PCAS patients at the British Columbia Cancer Agency from 1990 to 2006, and 9 PCAS patients at the Cleveland Clinic from 1988 to 2013. In addition, a total of 168 PCAS cases from 1973 to 2013 were documented in the SEER database. PCAS is a highly aggressive tumor that affects more males than females, with a mean age of onset of 44.4 ± 15.5 years old. The lungs, liver, brain, lymph nodes, bone, adrenal gland, and spleen are the most common sites of metastasis in the early stages. Besides, PCAS patients have a short overall survival time, with a reported median of 12-14 months abroad and a median of 3 months in China (5–8). In most cases, PCAS originates from the right atrium, presenting with cough, chest tightness, shortness of breath, palpitation, fever or weakness in the early stage, as well as other non-specific symptoms such as dyspnea and chest pain in the later stages (9). Metastatic lesions can induce various symptoms, including lumbago, which was the initial presentation of our patient.

Imaging is a valuable tool for diagnosing and evaluating PCAS. The ECG is the most basic non-invasive diagnostic tool available that can be used to detect around 75% of primary cardiac malignancies. Additionally, it provides critical information on valve function, tumor site, and the relationship between the tumor and the heart wall. Furthermore, ECG can also be used to assess cardiac function (10). In contrast, CT scans are often used for cancer diagnosis by revealing the presence of a cardiac space-occupying lesion, lesion enhancement using a contrast agent, lesion attachment to the myocardium, central necrosis and uneven density of the tumor, pericardial effusion, local invasion, and distant metastasis. Positron emission computed tomography (PET) is a non-invasive preoperative test that can identify primary lesions and potential metastases *via* their hypermetabolic properties (SUV value: 3.5-9.4). On the other hand, Cardiac magnetic resonance (CMR) can clearly reveal tumor information, including the anatomical location, size, tissue characteristics and the adjacent structures, which serve as an important reference for surgical assessment (5–11). Angiosarcoma manifestations on CMR include inhomogeneous isointense or long T1/T2 signals, mild patchy enhancement within the lesion at first and predominantly inhomogeneous enhancement during the delayed phase.

Cytology and IHC are the golden standards for the diagnosis of PCAS. Microscopically, well-differentiated PCAS has irregular vessel lumens and blood sinusoids of different diameters that are interconnected to form a mesh-like pattern. The lumens are filled with red blood cells and the inner walls are covered with tumor endothelial cells, which present with varying morphologies (spindle-shaped or epithelioid), significant atypia, abundant cytoplasm, large hyperchromatic nuclei with easily visible nuclear divisions (>10/10 HPF). In addition, local endothelial cells are hyperproliferative, manifesting as budding, protrusion or papilla. In contrast, poorly-differentiated PCAS presents with unremarkable vessel lumens with focal single-cell vessel lumen). The tumor cells are rounded or spindle-shaped, with a large nucleus and a prominent nucleolus, locally presenting with a spindle-shaped fibrosarcoma-like solid dense area. Meanwhile, surrounding tissues are infiltrated and damaged, partially involving the pericardium and accompanied by patchy hemorrhage and necrosis. Furthermore, IHC findings include diffuse enhancement of ERG, CD31, CD34, Vimentin, Fli-1 and FVIII, negative AE1/AE3, D2-40, SMA, MSA, MyoD1, Desmin, UEA-1, EMA, Myoglobin, Myosin, Calretinin, MC, WT-1, S-100 and HMB-45, and 30%-100% positive Ki-67 (12).

Given the small number of cases, there is no standardized treatment for PCAS to this date. Most medical facilities develop a personalized treatment regimen based on past experience. Therefore, there is an urgent need for evidence-based medicine. Surgery has been proven as an effective strategy to improve the prognosis of patients with localized PCAS. According to a British Columbia Cancer Agency analysis (4), the median OS in cases with resectable localized tumors is 25 months, which is substantially higher than the 6 months in non-resectable or non-R0 resections. Data from the French Sarcoma Group suggested that the median OS of localized PCAS patients receiving R0 resection, R1/R2 resection and those without surgical treatment was 38.8 months, 18.2 months and 11.2 months, respectively, with the extended survival attributed to tumor excision rather than adjuvant treatment (6). The Cleveland Clinic reported that the median OS was 67.6 months in surgical patients and 5.93 months in patients not undergoing surgery (7). In addition, the 1-, 3- and 5-year survival rates of angiosarcoma patients receiving surgical resection were 38.3%, 23.6% and 5.9%, respectively, compared to the 38.5%, 9.6% and 0% in patients not receiving surgical intervention (5). In some medical centers, heart transplantation is adopted as the main treatment strategy for PCAS; however, it was found that there was no significant superiority in terms of patient survival compared with palliative systemic therapy (9 months vs. 8 months) (13). Partial resection + extended resection + local reconstruction remains the standard procedure for PCAS. Nevertheless, R0 resection is difficult to achieve since neurovascular structures are often significantly affected by the tumor’s invasiveness, posing a serious challenge to cardiac surgery.

Neoadjuvant chemotherapy (NAC) is a viable alternative in the treatment of locally advanced PCAS patients that cannot undergo surgical resection. Radulescu et al (14) found that PCAS patients who received Adriamycin-based NAC had a median progression-free survival (PFS) of 33 months due to enhanced tumor resectability. In a cohort study comprising of 32 primary cardiac sarcoma patients (24 PCAS patients) scheduled for surgical treatment in the University of Texas Health Science Center at Houston from 1990 to 2005, NAC was conducted using Adriamycin plus Ifosfamide and Gemcitabine or Docetaxel if not tolerated. The results showed that the median OS in patients with and without NAC was 20 months and 9.5 months, respectively (15). Comparatively, palliative chemotherapy’s efficacy in advanced metastatic cases has yet to be validated by large-scale clinical trials. In the phase II ANGIOTAX trial (the only clinical trial on angiosarcoma), the objective remission rate (ORR) of patients (n=30) receiving Paclitaxel on a weekly regimen was 19%, the median PFS was 4 months and the median OS was 8 months (16). Apart from Paclitaxel, Anthracyclines, Gemcitabine, Ifosfamide, and Docetaxel are also clinically used alone or in combination. However, there were only a few case reports and retrospective studies and no randomized controlled trials to determine their efficacy. Furthermore, some targeted agents, primarily vascular endothelial growth factor (VEGF) -targeting agents such as Anlotinib, Bevacizumab, Sunitinib, and Pazopanib, are also used in PCAS treatment. Pazopanib was reportedly utilized in a case of metastatic PCAS, which resulted in complete remission following treatment (17). However, none of the agents have been subjected to large-scale clinical trials. In the present case, our patient received three cycles of Gemcitabine + Docetaxel and achieved SD. Notably, tumor markers such as CA125, NSE, and NT-proBNPa showed a consistent downward trend during treatment.

Due to the low incidence of angiosarcoma, large genomic studies have been challenging. Rosenbaum et al (18) performed genetic testing of 26 patients with angiosarcoma, the most common oncogenic or likely oncogenic abnormalities were TP53 alterations (27% of patients), MYC amplifications (23%), CRKL amplified (27%)and ATRX alterations (15%). Another genetic test that included the largest number of patients with angiosarcoma found the main mutation genes include TP53 (30%), KDR (26%), PIK3CA (21%), POT1 (19%), PLCG1 (17%), NF1 (13%), FAT1 (13%), NOTCH2 (13%), MYC (11%) and FGFR2, ATRX, BRAF, ARID1A, etc. (19). In addition, 33% of patients with angiosarcoma had recurrent amplification of the HOXA gene, which plays an important role in neovascularization and wound healing, suggesting a potential mechanism for HOXA amplification as an oncogene in angiosarcoma (20). Although more and more gene mutations have been found in angiosarcoma, but DNMT3A gene mutations have never been detected. The DNMT3A gene, located on chromosome 2p23, is made up of 26 exons and 25 introns and encodes a DNA methyltransferase that catalyzes the *de novo* transfer of methyl groups to specific CpG sites (21). DNMT3A is highly expressed by various hematopoietic stem cells and immune cells. Mutations in the DNMT3A gene can promote the initiation and development of atherosclerosis by inducing inflammatory reactions, and they can also increase myeloid differentiation and malignant transformation of bone marrow hematopoietic stem cells (HSCs) by interfering with their ability to self-renew (22, 23). Primary cardiac tumors are extremely rare. Angiosarcoma is the most common histologic subtype and is characterized by its permeability and destructive nature. Herein, our patient presented with a mutation in the DNMT3A gene, which draws attention to the role of DNMT3A mutation in the initiation and development of PCAS. Furthermore, whether PCAS and atherosclerosis share some pathophysiological processes and whether cardiovascular drugs are effective in this rare disease warrant further investigation.

## Conclusion

Primary cardiac angiosarcoma is an extremely uncommon tumor, to sum up. This is the first account of PCAS brought on by a DNMT3 gene mutation. It is frequently underdiagnosed or misdiagnosed due to its low prevalence and cryptic clinical appearance. Clinicians should include cardiac angiosarcoma in the initial differential diagnosis if there is even the slightest hint of suspicion. Due to its great sensitivity, echocardiography is one of the most effective diagnostic methods, and CT and MR scans are frequently utilized to determine whether metastases from other sites are present or not. Next-generation sequencing (NGS) and immunohistochemical staining are further diagnostic techniques. Contrarily, there aren’t any uniformly accepted clinical standards or reliable preventative strategies. Surgery is still the preferred method of treatment. Patients with advanced disease may benefit from chemotherapy, targeted therapy, or immunotherapy, but these treatments have variable therapeutic outcomes and poor prognoses. As a result, selecting the optimum course of treatment to significantly increase patient survival and quality of life is still a difficult decision that merits additional research. Our study emphasizes how challenging it is to identify and treat this uncommon type of malignancy.

## Data availability statement

The original contributions presented in the study are included in the article/**Supplementary Material**. Further inquiries can be directed to the corresponding author.

## Ethics statement

Written informed consent was obtained from the individual for the publication of any potentially identifiable images or data included in this article.

## Author contributions

KT, First authorship: contributed to editing, revising, and approving the manuscript. FS contributed to the writing and revision of the manuscript. YL, HZ, TW, TC, and XP contributed to collecting clinical data. All authors have read and approved the final manuscript.

## Funding

This work obtained funding support from the Gansu Province Science and Technology Department Key R&D Program (21JR1RA121), China.

## Conflict of interest

The authors declare that the research was conducted in the absence of any commercial or financial relationships that could be construed as a potential conflict of interest.

## Publisher’s note

All claims expressed in this article are solely those of the authors and do not necessarily represent those of their affiliated organizations, or those of the publisher, the editors and the reviewers. Any product that may be evaluated in this article, or claim that may be made by its manufacturer, is not guaranteed or endorsed by the publisher.

## References

[1] Butany J Nair V Naseemuddin A Nair GM Catton C Yau T. Cardiac tumours: diagnosis and management. Lancet Oncol (2005) 6(4):219–28. doi: 10.1016/S1470-2045(05)70093-0 15811617

[2] Antonuzzo L Rotella V Mazzoni F Doni L Bianchini D Garbini F. Primary cardiac angiosarcoma: a fatal disease. Case Rep Med (2009) 2009(591512):1–4. doi: 10.1155/2009/591512 PMC2731464 19724650

[3] Zhang PJ Brooks JS Goldblum JR Yoder B Seethala R Pawel B. Primary cardiac sarcomas: a clinicopathologic analysis of a series with follow-up information in 17 patients and emphasis on long-term survival. Hum Pathol (2008) 39(9):1385–95. doi: 10.1016/j.humpath.2008.01.019 PMC4081532 18602663

[4] Truong PT Jones SO Martens B Alexander C Paquette M Joe H. Treatment and outcomes in adult patients with primary cardiac sarcoma: the British Columbia cancer agency experience. Ann Surg Oncol (2009) 16(12):3358–65. doi: 10.1245/s10434-009-0734-8 19830494

[5] Zhang Z Cheng Y Gong X Ge Y Bai C Wang X. Characteristics and outcomes of primary angiosarcoma. Chin J Oncol (2019) 41(09):693–7. doi: 10.3760/cma.j.issn.0253-3766.2019.09.009 31550860

[6] Isambert N Ray-Coquard I Italiano A Rios M Kerbrat P Gauthier M. Primary cardiac sarcomas: a retrospective study of the French sarcoma group. Eur J Cancer (2014) 50(1):128–36. doi: 10.1016/j.ejca.2013.09.012 24135684

[7] Randhawa JS Budd GT Randhawa M Ahluwalia M Jia X Daw H. Primary cardiac sarcoma. Am J Clin Oncol (2016) 39(6):593–9. doi: 10.1097/COC.0000000000000106 25036471

[8] Zhang C Huang C Zhang X Zhao L Pan D. Clinical characteristics associated with primary cardiac angiosarcoma outcomes: a surveillance, epidemiology and end result analysis. Eur J Med Res (2019) 24(1):1–10. doi: 10.1186/s40001-019-0389-2 31426842 PMC6699122

[9] Luo L Zhao W Wang Y Liu K. Cardiac angiosarcoma: A case report and review of the literature. Echocardiography (2021) 38(12):2083–90. doi: 10.1111/echo.15221 34806222

[10] Kupsky DF Newman DB Kumar G Maleszewski JJ Edwards WD Klarich KW. Echocardiographic features of cardiac angiosarcomas: The Mayo clinic experience (1976-2013). Echocardiography (2016) 33(2):186–92. doi: 10.1111/echo.13060 26460068

[11] Krishnan T Pettersson G Mukherjee R Singhal N. Cardiac angiosarcoma: A diagnostic and therapeutic challenge. J Cardiol cases (2020) 22(2):90–3. doi: 10.1016/j.jccase.2020.04.010 PMC7403564 32774528

[12] Teng F Chen D Fang H Shang J Wu Y Cui Y. Clinicopathology analysis of 9 cases in primary cardiac angiosarcoma. J Cardiovasc pulmonary Dis (2016) 35(12):974–7. doi: 10.3969/j.issn.1007-5062.2016.12.011

[13] Li H Yang S Chen H Yang Z Hong T Hou Y. Survival after heart transplantation for non-metastatic primary cardiac sarcoma. J cardiothoracic Surg (2016) 11(1):145. doi: 10.1186/s13019-016-0540-x PMC5048623 27716444

[14] Radulescu D Pripon S Radulescu LI Constantea NA Gulei I. A rare case of primitive right atrium angio-sarcoma with favorable outcome, in a young female. Case Rep literature review Rev Med Chile (2008) 136(10):1311–6. doi: 10.4067/S0034-98872008001000012 19194629

[15] Blackmon SH Reardon MJ. Surgical treatment of primary cardiac sarcomas. Texas Heart Institute J (2009) 36(5):451–2. PMC2763478 19876427

[16] Penel N Bui BN Bay JO Cupissol D Ray-Coquard I Piperno-Neumann S. Phase II trial of weekly paclitaxel for unresectable angiosarcoma: the ANGIOTAX study. J Clin (2008) 26(32):5269–74. doi: 10.1200/jco.2008.17.3146 18809609

[17] Schur S Hamacher R Brodowicz T. Pazopanib in primary cardiac angiosarcoma of the right atrium: A case report. Case Rep Oncol (2016) 9(2):363–7. doi: 10.1159/000447088 PMC4939669 27462238

[18] Rosenbaum E Antonescu CR Smith S Bradic M Kashani D Richards AL. Clinical, genomic, and transcriptomic correlates of response to immune checkpoint blockade-based therapy in a cohort of patients with angiosarcoma treated at a single center. J Immunother Cancer (2022) 10(4):e004149. doi: 10.1136/jitc-2021-004149 35365586 PMC8977792

[19] Painter CA Jain E Tomson BN Dunphy M Stoddard RE Thomas BS. The angiosarcoma project: enabling genomic and clinical discoveries in a rare cancer through patient-partnered research. Nat Med (2020) 26(2):181–7. doi: 10.1038/s41591-019-0749-z 32042194

[20] Xie HM Bernt KM. HOXA amplification defines a genetically distinct subset of angiosarcomas. Biomolecules (2022) 12(8):1124. doi: 10.3390/biom12081124 36009018 PMC9406048

[21] Cole CB Russler-Germain DA Ketkar S Verdoni AM Smith AM Bangert CV. Haploinsufficiency for DNA methyltransferase 3A predisposes hematopoietic cells to myeloid malignancies. J Clin Invest (2017) 127(10):3657–74. doi: 10.1172/jci93041 PMC5617681 28872462

[22] Cobo I Tanaka T Glass CK Yeang C. Clonal hematopoiesis driven by DNMT3A and TET2 mutations: role in monocyte and macrophage biology and atherosclerotic cardiovascular disease. Curr Opin Hematol (2022) 29(1):1–7. doi: 10.1097/moh.0000000000000688 34654019 PMC8639635

[23] Guryanova OA Shank K Spitzer B Luciani L Koche RP Garrett-Bakelman FE. DNMT3A mutations promote anthracycline resistance in acute myeloid leukemia *via* impaired nucleosome remodeling. Nat Med (2016) 22(12):1488–95. doi: 10.1038/nm.4210 PMC5359771 27841873

