# Supplementary material for: A case report of primary cardiac angiosarcoma with DNMT3A gene mutation
Source: Front Oncol. 2022 Oct 27;12:1018741. doi: 10.3389/fonc.2022.1018741 (PMC9647023; doi:10.3389/fonc.2022.1018741)
Supplement: Supplementary file 1 [file DataSheet_1.docx]

CARE Supplementary materials

**Patient Information** **5c：**Medical, family, and psycho-social history including relevant genetic information：The patient was previously fit and healthy, with no specific family history and no specific cancer pedigree was found.

**Timeline 7：**Historical and current information from this episode of care organized as a timeline

1. presented to the Orthopaedics Department of our hospital complaining of "lumbago for a week" in early March 2020. To clarify the diagnosis, also start the following inspections in ②③④⑤.
2. CT imaging revealed bone destruction at the L3 vertebral level, A biopsy was conducted, and pathological analysis revealed angiosarcoma (L3).
3. a whole-body CT scan was performed. Space-occupying lesions in the right atrium, liver segment S8, pericardium, spleen and bone, as well as pericardial effusion, right pleural effusion and incomplete right lower lung distension were detected.
4. Echocardiography (ECG) revealed: a hypoechoic lesion (6.4×5.7 cm) in the right atrial roof with inhomogeneous echogenicity causing compression of the atrium.
5. because of patient experienced pericardiac tamponade, Pericardiocentesis and pericardial drainage were performed, screened for cancer cells using cytology.
6. The patient was diagnosed with an advanced stage of PCAS
7. next-generation sequencing (NGS) revealed a 10.7% abundance of a missense mutation in exon 19 of the DNMT3A gene (p.Glu733Ala).
8. The patient received Gemcitabine (1.4 g, Day 1, 8) and Docetaxel (150 mg, Day 8) for one cycles starting May 2020.
9. after 1 cycle, stable disease (SD) was achieved on CT scan
10. The patient received Gemcitabine (1.5g, Day 1, 8) and Docetaxel (150 mg, Day 8) for one cycles starting June 2020.
11. in mid-July, the patient presented with acute chest pain, with blood pressure fluctuating between 80-90 and 40-50 mmHg. CT scan indicated significant pleural effusions (See Fig. 3), and a routine blood test revealed an RBC of 2.69*10-12/L and HGB of 76 g/L.
12. the patient subsequently died three days later.
